# Early and Longitudinal Humoral Response to the SARS-CoV-2 mRNA BNT162b2 Vaccine in Healthcare Workers: Significance of BMI, Adipose Tissue and Muscle Mass on Long-Lasting Post-Vaccinal Immunity

**DOI:** 10.3390/v14050868

**Published:** 2022-04-22

**Authors:** Marlena Golec, Martyna Fronczek, Joanna Zembala-John, Martyna Chrapiec, Adam Konka, Karolina Wystyrk, Hanna Botor, Zenon Brzoza, Sławomir Kasperczyk, Rafał Jakub Bułdak

**Affiliations:** 1 Silesian Park of Medical Technology Kardio-Med Silesia, M. Curie-Skłodowskiej 10C, 41-800 Zabrze, Poland; mfronczek@sum.edu.pl (M.F.); jzembala@sum.edu.pl (J.Z.-J.); m.chrapiec@kmptm.pl (M.C.); a.konka@kmptm.pl (A.K.); k.wystyrk@kmptm.pl (K.W.); 2 Department of Pharmacology, Faculty of Medical Sciences in Zabrze, Medical University of Silesia in Katowice, H. Jordana 38, 41-808 Zabrze, Poland; 3 Department of Medicine and Environmental Epidemiology, Faculty of Medical Sciences in Zabrze, Medical University of Silesia in Katowice, H. Jordana 19, 41-808 Zabrze, Poland; 4 Silesian Center for Heart Diseases in Zabrze, M. Curie-Skłodowskiej 9, 41-800 Zabrze, Poland; 5 Acellmed Ltd., M. Curie-Skłodowskiej 10C, 41-800 Zabrze, Poland; h.botor@acellmed.pl; 6 Department of Internal Diseases, Allergology, Endocrinology and Gastroenterology, Institute of Medical Sciences, University of Opole, Al. W. Witosa 26, 40-451 Opole, Poland; zenon.brzoza@uni.opole.pl; 7 Department of Biochemistry, Faculty of Medical Sciences in Zabrze, Medical University of Silesia in Katowice, H. Jordana 19, 41-808 Zabrze, Poland; skasperczyk@sum.edu.pl; 8 Department of Clinical Biochemistry and Laboratory Diagnostics, Institute of Medical Sciences, University of Opole, Oleska 48, 45-052 Opole, Poland

**Keywords:** IgG antibody, SARS-CoV-2, COVID-19, healthcare workers, health care professionals, COVID-19 vaccine, BNT162 vaccine, adipose tissue, body composition, immunization

## Abstract

Background: This study aimed to investigate the early and longitudinal humoral response in Healthcare Workers (HCWs) after two doses of the BNT162b2 vaccine and to assess the association between metabolic and anthropometric parameters and the humoral response after vaccination. Methods: The study included 243 fully vaccinated HCWs: 25.50% previously infected with SARS-CoV-2 (with prior history of COVID-19—PH) and 74.40%—uninfected, seronegative before the first vaccination (with no prior history of COVID-19—NPH). IgG antibodies were measured, and sera were collected: prior to the vaccination, 21 days after the first dose, and 14 days and 8 months after the second dose. Results: 21 days after the first dose, 90.95% of individuals were seropositive; 14 days after the second dose, persistent immunity was observed in 99.18% HCWs, 8 months after complete vaccination—in 61.73%. Statistical analysis revealed that HCWs with PH had a greater chance of maintaining a humoral response beyond eight months after vaccination. Increased muscle mass, decreased fat mass, and younger age may positively affect long-term immunity. Smokers have a reduced chance of developing immunity compared to non-smokers. Conclusions: Fully vaccinated HCWs with PH are more likely to be seropositive than fully inoculated volunteers with NPH.

## 1. Introduction

Coronavirus disease 2019 (COVID-19) is an infectious zoonotic illness caused by a novel pathogen belonging to the broad and diverse family of *Coronaviridae*, subfamily *Orthocoronavirinae*, genus *Betacoronavirus* [1].

The first case of SARS-CoV-2 infection was detected in December 2019 in Wuhan, Hubei Province, China. Two years after its emergence, the total number of confirmed SARS-CoV-2 infections exceeded 271 million, and the number of deaths—5.3 million [2]. High hopes to stop the spread of SARS-CoV-2 were placed in global vaccination against COVID-19.

The first vaccines were administered in December 2020; as the vaccination program progressed, a temporal stabilization or even a downward trend in the range of newly recorded weekly COVID-19 cases and deaths was observed in many countries [3]. However, data on the durability and effectiveness of humoral response after vaccination are still scarce. Furthermore, knowledge about factors affecting long-term immunity remains insufficient. There is increasing evidence that certain factors and conditions, e.g., advanced age, male gender, visceral adiposity, or obesity, are associated with a higher risk for severe COVID-19 outcomes [4,5,6]. Factors affecting one’s immunity against SARS-CoV-2 and its durability, however, are still being investigated.

Serological tests constitute a crucial element of SARS-CoV-2 infection prevention and control strategies. They provide an exemplary method for assessing the incidence of coronavirus infection and monitoring seroconversion and seroversion in the population [7,8,9]. By measuring antibody levels, it is possible to observe the dynamics of specific antibody responses after vaccination against COVID-19. It is assumed that the prevalence of particular serum antibodies (IgG and IgM) to SARS-CoV-2 can be used to assess exposure to SARS-CoV-2 in the population [10,11]. From an epidemiological point of view, serological surveillance of anti-SARS-CoV-2 IgG antibody levels in the population could also be used to estimate the cumulative COVID-19 incidence/exposure rate [11,12].

Routine serological testing takes on particular meaning in the context of healthcare workers (HCWs)—a group requiring a special concern and protection during the pandemic, for numerous reasons. First, as the front-line workers combat COVID-19, HCWs are at higher risk of contracting SARS-CoV-2 and transmitting the virus to others (workmates and patients) than other professional groups [13]. The risk rises even more in cases of asymptomatic or pre-symptomatic infection, occurring in up to 30–40% of individuals [14]. There is a growing body of evidence that the silent transmission of SARS-CoV-2 constitutes a major contributor to the COVID-19 pandemic [15]. Second, having in mind a shortage of medical staff in general, it has become critical to maintaining the healthcare workforce on the board. Each HCW’s absence at work (due to COVID-19 isolation or quarantine) poses a significant, additional threat in maintaining the efficiency of the healthcare system and providing effective care of COVID-19 and non-COVID-19 patients. Finally, compliance with safety protocols and the use of protective equipment alone are insufficient to protect this group from infection [16]. Therefore, vaccination and monitoring of the durability of the immune response constitute an essential part of the prevention and infection-control strategy in this group.

### Aims

This prospective, observational single-center study aimed to assess early and longitudinal antibody response after COVID-19 vaccination among HCWs on the timeline: after receiving the first and second dose of BNT162b2 mRNA vaccine (Comirnaty; BioNTech/Pfizer, New York, NY, USA). Moreover, the study aimed to investigate a potential correlation between selected factors, such as gender, age, body mass index (BMI), smoking, body composition, being convalescent, and early- and long-term humoral response in this group.

Furthermore, we tested the hypothesis that decreased fat tissue and increased muscle mass tissue may positively affect long-term immunity in fully vaccinated individuals.

## 2. Materials and Methods

### 2.1. The Study Group

**Figure 1:**
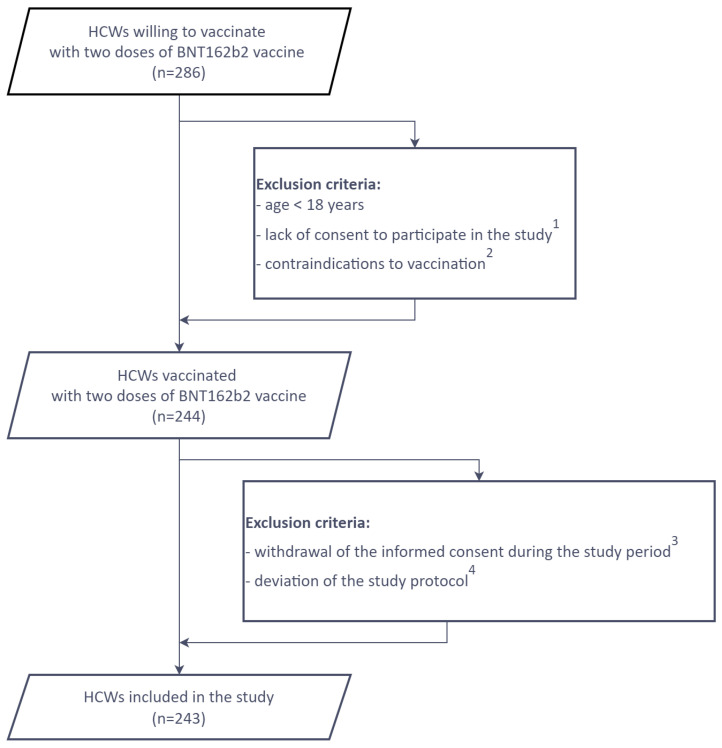
Flowchart of study selection based on the inclusion and exclusion criteria. Legend: 1—lack of one’s consent to participate in the study after becoming acquainted with the study informed consent form and planned four blood collection schedule (Figure 2); 2—medical contraindications to vaccination; 3—withdrawal of the informed consent during the study period for personal or other reasons; 4—deviation of the study protocol (i.a., not being able to participate at the blood collection according to predicted blood collection schedule). Abbreviations: HCWs—healthcare workers.

The study was conducted among HCWs working in a Regional Specialized Hospital no. 4 in Bytom in Upper Silesia—one of the Polish regions most severely affected by COVID-19. Before collecting the data and specimens, all participants provided their written informed consent. The study was approved by the Institutional Review Board of the Medical University of Silesia in Katowice (PCN/0022/KB1/50/20) and was conducted in accordance with the Helsinki Declaration. The inclusion and exclusion criteria are presented below (Figure 1).

**Figure 2:**
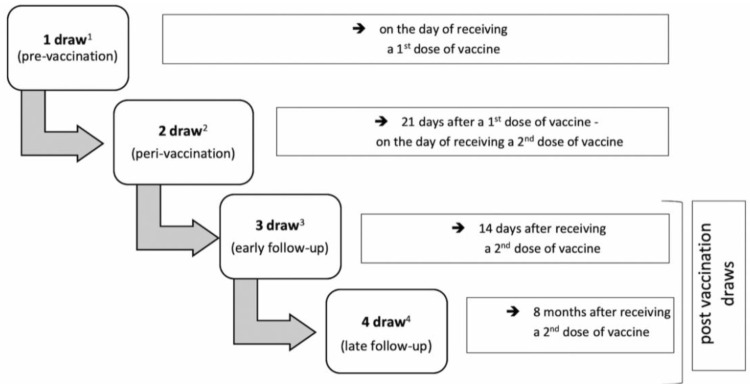
Schematic diagram of the sample collections. 1—draw before starting the vaccination (on the day of receiving the first dose of the vaccine); 2—draw on the day of receiving the second dose of the vaccine (21 days after receiving the first dose); 3—draw 14 days after receiving the second dose of the vaccine; 4—draw 8 months after receiving the second dose of the vaccine. The first two blood draws were performed prior to administering the vaccine.

The vaccination program in the study group was conducted from 29 December to 15 January 2021 (first dose), from 20 January to 11 February 2021 (second dose). Following the study protocol, participants underwent four blood draws: two during the vaccination process, and two—post-vaccination (the third and fourth draw was performed from 3 February to 25 February 2021, and from 27 September to 8 October 2021, respectively) (Figure 2). According to the official epidemiological data, during the first three blood collections, the dominant variant of SARS-CoV-2 in the region of Upper Silesia was Alpha (B.1.1.7 variant), and during the fourth blood draw—Delta (B.1.617.2 variant) [17,18].

### 2.2. Participants’ Characteristics

Participants’ demographics (age, gender, position, workplace—type of unit/department) and primary clinical data: smoking, self-reported presence of chronic diseases (including high risk for COVID-19 conditions, such as: diabetes, autoimmune diseases, hypertension, chronic cardiovascular diseases, chronic obstructive pulmonary disease, asthma, chronic kidney disease, cancers, and other) and COVID-19 status before the study start (i.e., with prior history of COVID-19—PH/with no prior history of COVID-19—NPH/unknown), symptomatic vs. asymptomatic course of the disease) were obtained through an author questionnaire.

### 2.3. Anti-SARS-CoV-2 IgG Measurement

Serum samples were analyzed using ACCESS SARS-CoV-2 (Beckman Coulter Inc., Brea, CA, USA)—a two-step enzyme chemiluminescent immunoassay (CLIA), according to the manufacturer’s instructions [19]. This assay helps to identify subjects with an adaptive immune response to SARS-CoV-2, indicating recent or prior infection. It detects IgG antibodies to the Receptor-Binding Domain (RBD) of the SARS-CoV-2 spike protein (S1 protein). In this study, IgG against RBD of S1 protein was detected in a semi-quantitative assay with a lower limit of quantitation (LoQ) of 2.00 AU/mL (arbitrary units/mL) and an upper limit for quantitative evaluation at 8000.00 AU/mL. According to the manufacturer’s results interpretation guidelines, the test samples were considered reactive for SARS-CoV-2 IgG antibodies when their value was ≥10.00 AU/mL and non-reactive when the test value was <10.00 AU/mL.

### 2.4. Body Composition Measurement Using the TANITA Analyzer

Excessive body mass has been considered one of the main risk factors for severe COVID-19 infection. To investigate whether it also impacted the level of humoral response, an analysis of potential correlations between those variables was conducted. Anthropometric parameters were measured, and body composition was analyzed using the TANITA Body Composition Analyzer MC-780MA (TANITA Corporation, Tokyo, Japan). This non-invasive device, using an electrical bioimpedance analysis (BIA), is certified and approved for clinical use, with NAWI CLASS III standards for scales used for medical measurements, EU certification CE0122. It also complies with the Medical Device Directive (MDD) 93/42/EEC for medical devices.

Participants with a physical disability, those pregnant, or those not consenting to undergo this part of the study, were excluded from the measurements. Body mass and height were measured, and BMI (Quetelet’s index) was assessed for consenting participants. It was calculated as a person’s weight in kilograms divided by the square of the person’s height in meters. Its results were interpreted according to the WHO classification: <18.50 means underweight, 18.50–24.99—normal body mass, 25.00–29.99—overweight, and ≥30.00—obesity [20]. In addition, waist circumference (WC) and hip circumference (HC) were measured: WC was measured at the midpoint between the lower margin of the last rib and the top of the iliac crest and hip circumference and HC—around the widest portion of the buttocks. The measurements were used to calculate the waist-to-hip ratio (WHR), using the formula: WC (cm)/HC (cm).

Moreover, basal metabolic rate (BMR) was assessed based on weight and age parameters. Before the examination, the participant’s data (identification number, gender, date of birth, height, body type - standard vs. athletic) and clothing weight were entered into the GMON software (GMON Pro 3.4.5, Medizin&Service GmbH, Chemnitz, Germany). During the analysis, the examined individual was asked to stand in the appropriate position (still, arms straight down) and bare feet on the TANITA platform. Body composition was assessed for body fat mass (BFM), fat-free mass (FFM), visceral fat (VFAT), predicted muscle mass (PMM), and total body water (TBW). The adipose tissue and muscle mass analyses were performed separately for each arm, leg, and trunk. Five different impedance results of the right arm, right leg, left arm, left leg, and trunk were then quantified. Measurements were made following the device manufacturer’s instructions. They were performed by the same study team members during the fourth blood collection, eight months after the second vaccine dose.

### 2.5. Statistical Analysis

Data were presented as the mean ± standard deviation for quantitative variables and counts and % values for qualitative variables. The normality of the distribution in the individual groups was assessed using a Q-Q plot. It was observed that for IgG titers in individual measurements, the distribution of the feature was right-skewed, and a better fit to the normal distribution was obtained through logarithmic transformation. Repeatable measures ANOVA was used to compare changes in IgG titers over time. The assumption of sphericity was verified using the Mauchly test; in case of failure, the Greenhouse–Geisser correction was applied. A paired t-test with the Bonferroni–Holm correction was used for post hoc comparisons. The comparison of pairs of variables was performed using the Student’s t-test to assess the homogeneity of variance using the Brown–Forsythe test. Comparisons of more than two groups at the one-time point were performed using a one-way analysis of variance with Tukey’s post hoc test. The relationship between the quantitative variables was analyzed using the Pearson correlation coefficient. A logistic regression multivariable model was built to assess the influence of examined variables on the chance of maintaining long-term seropositivity. The model was built using a stepwise backward method and presented as odds ratio (OR) and its 95% confidence interval (CI). *p* values < 0.05 were considered significant. The R language with the GGplot library was used to analyze [21,22].

## 3. Results

Initially, 286 HCWs vaccinated with two BNT162b2 doses were enrolled in the study. Eventually, 243 individuals who met the inclusion criteria were included: 187 (76.95%) women and 56 men (23.05%). The mean age in this group was 47.42 years (range: 22.00–66.00 years). The demographic and clinical characteristics of the study cohort, as well as the TANITA results, are presented below (Table 1).

Of the study cohort, 62 HCWs (25.51%) were seropositive (IgG ≥ 10.00 AU/mL) before the first dose of the vaccine. Immediately before administration of the second dose of vaccine (21 days after receiving the first dose), 221 participants (90.95%) had IgG antibody levels ≥ 10.00 AU/mL. 14 days after receiving the second dose of vaccine, 241 participants (99.18%) were identified as seropositive.

Fourteen days after receiving the second dose of the vaccine (early follow-up), a high titer of IgG (>350.00 AU/mL) was determined in 148 participants (60.91%), low—only in 3 individuals (1.23%). However, after eight months, a high titer of the IgG (>350.00 AU/mL) was detected only in 1 HCW (0.41%), while in 93 (38.27%) of the subjects, the level of antibodies was determined as seronegative (<10.00 AU/mL). Long-term follow-up indicated persistent immunity (IgG > 10.00 AU/mL) in 150 HCWs (61.73%). Antibody titers at each time interval are presented in Table 2.

The correlation of anthropometric parameters with IgG antibody titer after 14 days from full vaccination or IgG antibody titer after 8 months from second vaccine dose was also assessed using the Pearson correlation method. Due to the lack of a significant linear relationship between the studied variables, the data were not shown. To identify factors affecting immunity levels after eight months, firstly, a one-way logistic regression was performed. Its results indicated that being seropositive at the study baseline (i.e., being a convalescent) increases the 8-fold chances of developing a long-term humoral response to COVID-19 (OR 8.64, 95% CI 3.54–21.06, *p* = 0.00). On the other hand, an age above 60 years reduces the chances of maintaining the appropriate amount of IgG antibodies after an 8-month follow-up (OR 0.30, 95% CI 0.14–0.64, *p* = 0.00). The performed analysis did not confirm the influence of such factors as gender, smoking, BMI, obesity, BFM, FFM, TBW, PMM, bone mass, impedance, or BMR on the chance of maintaining long-lasting immunity to SARS-CoV-2 (*p* > 0.05) (Table 3).

**Figure 3:**
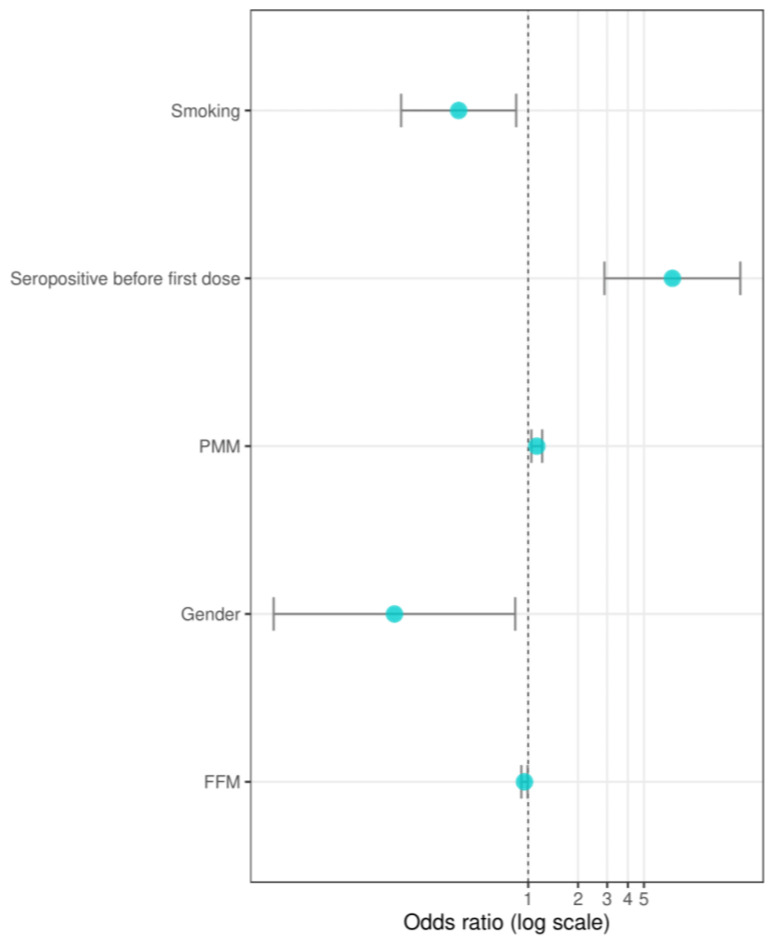
Association between selected parameters of IgG seropositivity. Odds ratio (OR) calculated with logistic regression. The figure presents the results as the mean ± 1.96 × standard error (95% confidence interval). Abbreviations: PMM—predicted muscle mass, FFM—fat-free mass.

Then, to confirm those observations and verify factors affecting the titer of anti-SARS-CoV-2 IgG antibodies (such as gender, undergoing COVID-19 before the study start, smoking, level of adipose tissue mass, and muscle mass), a multivariate analysis using a stepwise backward method was performed (Figure 3).

The results of this analysis revealed that HCWs with PH before receiving the first dose of BNT162b2 vaccine had a 7-fold greater chance of maintaining a humoral response beyond eight months after a complete, two-dose vaccination course (OR 7.43, 95% CI 2.89–19.11, *p* = 0.00). Gender impacted the chance of maintaining long-term immunity after full vaccination (OR 0.16, 95% CI 0.03–0.83, *p* = 0.03). Male gender was associated with a lower chance of preserving immunity after eight months, whereas females were 6-times more likely to keep the long-lasting immunity. In addition, our research demonstrated that both age and body composition (BFM, PMM) impact the durability of one’s humoral response and are good predictors of long-term immunity. PMM was an additional parameter that positively affected the long-term immune response. An increase in PMM by one kg raised the chance of maintaining a long-term immunity (OR 1.13, 95% CI 1.04–1.21, *p* = 0.00), whereas an increased BFM reduced the chance of longitudinal immune response (OR 0.95, 95% CI 0.91–0.99, *p* = 0.02). Furthermore, smokers also had a lower chance of developing long-lasting immunity after vaccination than non-smokers (OR 0.38, 95% CI 0.17–0.85, *p* = 0.02).

Additionally, anthropometric parameters were compared between the seronegative and seropositive groups at the fourth time point of the study. There was a significant difference in right-hand FFM (2.56 ± 0.70 vs. 2.76 ± 0.88, *p* = 0.05), left-hand FFM (2.56 ± 0.73 vs. 2.78 ± 0.91, *p* = 0.04), and left-hand PMM (2.44 ± 0.69 vs. 2.63 ± 0.85, *p* = 0.05) between seronegative and seropositive individuals. In addition, we performed correlation analyses between BFM and PMM in the study population, in the group of seropositive and seronegative HCWs, also divided into right and left arms. The results indicated a significant weak positive correlation in each of the analyzed subgroups (0.24 ≥ r ≤ 0.44; *p* < 0.05). Data not included in the manuscript.

**Figure 4:**
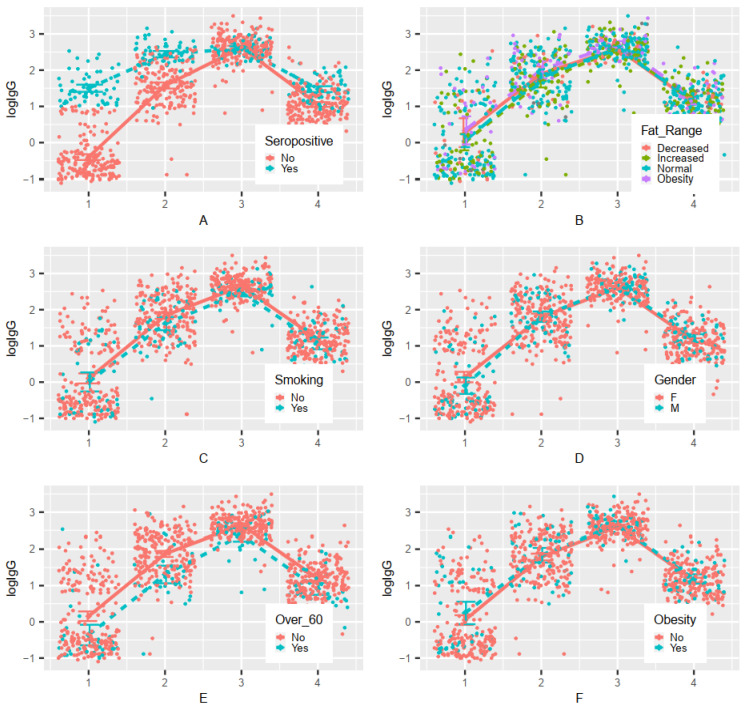
Changes in IgG titer over time depending on selected parameters (ANOVA results) before the first dose (1), before the second dose (2), early FU 14 days after receiving the second dose (3), late FU eight months after full inoculation (4): A—COVID-19 disease before first vaccination: significant time and group effect and interaction of the time and group *p* < 0.05; B—fat range: significant time effect *p* < 0.05; C—smoking: significant time effect *p* < 0.05; D—gender: significant time effect and interaction of time and group *p* < 0.05; E—age over 60: significant time and group effects *p* < 0.05; F—obesity: significant time effect *p* < 0.05. Abbreviations: seropositive ≥ IgG 10.00 AU/mL; fat range: decreased—BMI < 18.50, increased—BMI 25.00–19.00, normal—BMI 18.50–24.99, obesity—BMI ≥ 30.00, F—female, m—male.

The performed analysis of variance (ANOVA) revealed that before the first vaccination, at the study baseline, HCWs with PH had a naturally higher antibody titer than those with NPH; this phenomenon was also observed before the administration of the second dose (peri-vaccination draw). Fourteen days after the second dose, however, the maximum level of IgG in those two groups was comparable. Interestingly, eight months after the second dose, HCWs with PH again achieved significantly higher antibody titers than NPH volunteers. No significant interaction between antibody titer on the timeline and adipose tissue level was observed. Regardless of one’s fat tissue range, after the first and second dose of vaccine (early follow-up), a similar increase in antibody levels had been reported, and eight months after complete vaccination (late follow-up)—a similar decrease in IgG levels. Furthermore, smoking had no significant effect on the process of developing immunity after vaccination. Moreover, there was no significant effect of gender on changes in antibody titer after vaccination. Participants over 60 years of age had a similar chance of developing immunity as those under 60 years of age; no significant age-related interaction was observed. However, it was noted that the antibody titer in the group over 60 years was lower than in the younger cohort at each measurement point. In addition, there was no significant effect of obesity on the course of immunization after vaccination (Figure 4).

## 4. Discussion

Vaccination against SARS-CoV-2 has been considered one of the most effective strategies to keep the spread of the virus under control. Although since the beginning the fast-track of vaccine development, a short path from the lab to the market, has aroused many controversies among the public worldwide, epidemiological data confirm that inoculation against COVID-19 results in a lower number of hospitalizations and deaths.

Vaccination against SARS-CoV-2 is highly recommended, especially in groups with high exposure risk, such as frontline responders or healthcare workers. In response to those guidelines, many countries, such as France, Greece, and Italy, have introduced mandatory vaccinations against COVID-19 for HCWs [23].

Published systematic reviews and meta-analyses show a significant effect of vaccination against SARS-CoV-2 on the significant prevention of symptomatic and asymptomatic COVID-19 among HCWs [24,25,26,27]. A meta-analysis by Chandan et al. points to a reduced incidence of COVID-19 infection and reduced hospitalizations and deaths among vaccinated healthcare professionals. It should be highlighted, however, that the authors themselves emphasized that the data obtained from the publications that were used to prepare the meta-analysis showed great heterogeneity. The collected literature data differed in terms of, i.a., the type of vaccine used, the number of HCWs, or the ethnic origin of the respondents. Additionally, most of the studies found were retrospective in nature, which could have contributed to misselection [25]. Another published meta-analysis showed that COVID-19 vaccines are effective after just one dose. However, the authors themselves emphasize that further observational studies are needed to assess the effectiveness of the vaccine against new variants of SARS-CoV-2 [26]. Published studies support the importance of vaccination among HCWs but emphasize that further research is needed to determine the duration of vaccine-induced protection among healthcare workers [24].

The primary objective of our study was to investigate early and late humoral responses following the administration of the BioNTech/Pfizer BNT162b2 COVID-19 vaccine. Our research demonstrated that 14 days after receiving the second dose of vaccine and finishing the complete vaccination course, almost all, i.e., 241 out of 243 HCWs (99.18%), became seropositive. These results indicate a vital immune system stimulation in response to secondary contact with the mRNA vaccine, manifested by increased IgG anti-SARS-CoV-2 antibody levels. These observations confirm the efficacy of vaccination and are in accordance with Liu et al.’s results presented in their systematic review and meta-analysis [28].

However, our long-term follow-up results revealed a significant waning of the humoral response over time. Eight months after complete vaccination, only 150 individuals (61.73%) remained seropositive, which suggests a reduction in activation of B lymphocytes stimulated by SARS-CoV-2 virus S protein. A similar trend was observed in the study conducted by Levin et al., where they found a substantial decline in humoral response in study participants six months after receiving the second dose of the vaccine, noticeable especially among men, subjects aged 65 years or older, and those with immunosuppression [29]. Additionally, Novello et al., in their study, indicated that although immunity to COVID-19 was maintained in the examined group at the long-term follow-up, it was waning with time [30].

Our study also investigated a potential correlation between selected variables and humoral response following vaccination to identify which factors affect vaccination-induced immunity the most.

Numerous studies indicate that gender affects immune system functions and that the female sex is associated with a more robust, faster, and more effective innate and adaptive (both humoral and cellular) immune response [31,32,33,34]. Gender-related differences in immune responses make men and women susceptible to infectious diseases in varying degrees. Moreover, they also affect one’s post-vaccinal immunity [35]. In our study using multivariate logistic regression analysis, we found that the female gender was associated with a 6-times greater chance of developing long-term immunity than males. However, it should be noted that our study group was strongly feminized, which might have affected our analysis results. Interestingly, when conducting an ANOVA, we did not observe an apparent difference in SARS-CoV-2 specific IgG titers between genders. Similarly, Levin et al. found that post-vaccinal immunity in women was higher than in men [29]. We also noted that age affects different humoral responses between men and women.

The adverse effect of age on the COVID-19 course and prognosis has already been reported in numerous studies [36,37]. According to Powell et al., in the United States, 80% of deaths from COVID-19 occur in people over the age of 65 [38]. Age-related changes in the body’s innate and adaptive immune function result in increased susceptibility to infections, including COVID-19, among the elderly. This phenomenon is related to cell aging and the influence of the environment on the proliferation and differentiation of immune cells in response to antigenic stimulation [32].

Our research revealed that people over 60 years of age have a lower chance of developing long-term immune responses. These observations are consistent with other studies investigating the correlation between age and COVID-19 vaccine-induced immunity. Levin et al., in their study on a large cohort of 4868 HCWs, found a significant association between the durability of the humoral response and age; six months after the second doses of BNT162b2 vaccine, older individuals had significantly lower IgG titers than their younger counterparts [29]. Likewise, Amodio et al. reported a significant reduction in anti-SARS-CoV-2 IgG levels in older vaccinees; this phenomenon was noticeable, especially in older men [39]. Additionally, Naaber et al. confirmed a negative correlation between IgG antibodies to the SARS-CoV-2 Spike protein receptor-binding domain (S-RBD) and the age of the vaccinated with mRNA BNT162b2 vaccine subjects. Their study indicated a weaker humoral response in older individuals both after the first and the second dose of vaccine, and a more rapid waning of antibodies after inoculation in this group [40]. On the other hand, they reported that age had a less significant effect at later time points after complete vaccination (i.e., in the case of their study, 3 and 6 months after the second dose). An age-dependent negative correlation in S1-specific IgG for SARS-CoV-2 following vaccination with mRNA BNT162b2 vaccine was also reported by Müller et al. The results of their study on differences in the immune response to the first and second dose of the vaccine between subjects younger than 60 years and older than 80 years indicated that older vaccinees had significantly lower IgG antibody titers than their younger counterparts [41]. The arguments cited above prove the usefulness of vaccination against SARS-CoV-2, especially among the elderly, even if the effectiveness of immunization diminishes with age.

Conducted in our study, one-way logistic regression analysis did not confirm the influence of BMI, obesity, and body composition (BFM, FFM, TBW, PMM, bone mass, and BMR) on the chances of developing a long-lasting humoral response. However, different conclusions could be drawn from a multivariable analysis performed using backward stepwise regression. Using this method, we did not eliminate the variables manually, but allowed the computer to adjust the predictors optimally for the best model. The obtained results revealed that both age and selected body composition parameters (fat and muscle mass) affect the humoral response of tested HCWs, and could be considered good predictors of long-lasting immunity. Our findings suggest that in younger volunteers, increased muscle mass and decreased adipose tissue levels could enhance the effectiveness and durability of immunization. Additionally, seronegative HCWs had lower FFM in the right and left arms and lower left arm PMM compared to seropositive subjects. This observation is similar to the previous results showing that greater PMM has an impact on the process of long-term immunization. Due to the fact that this value was on the border of the significance of the test, further analysis should be conducted with the participation of a larger number of people.

The current evidence shows that severe COVID-19 course in some individuals is associated with “a cytokine storm”, which could be a consequence of increased secretion of pro-inflammatory cytokines directly by adipose tissue of obese subjects. On the other hand, muscle cachexia (increased muscle protein catabolism) or malnutrition, common in older people, may negatively affect the vaccine-induced immunization process [42].

Skeletal muscles are increasingly recognized as organs with endocrine properties. They regulate the function of the immune system through myokine signaling and the expression of surface molecules that modulate the immune system [43]. It has been shown that skeletal muscle homeostasis may be partly responsible for the proper functioning of the immune system. Therefore, a decline in muscle mass that disrupts skeletal muscle homeostasis may compromise the immune system. Furthermore, it has been suggested that the biological aging of the organism related, i.a., to the lack of physical activity and metabolic changes, as well as a decrease in muscle mass, affects the proper functioning of the muscular system, which results in impaired immune function of the body [43]. Our analysis showed that a single change in muscle tissue increased the chances of being seropositive after two vaccine doses. It follows that “the vaccine better immunizes metabolically young” people with a higher content of muscle tissue. Our multivariate analysis results demonstrated that the chance of maintaining long-lasting immunity seems to be better described by the model containing the mass of adipose tissue and muscle mass than the age criterion.

The term ‘obesity’ describes the accumulation of excessive amounts of BFM in adipose tissue, subcutaneous tissue, or within selected organs [44]. The main problem in understanding whether obesity does affects one’s immunity and to what extent is that the obese population is heterogeneous regarding overall health profile, presence of potential co-morbidities, dietary patterns, and individual microbial and social environments [45]. From an anatomical and metabolic perspective, this term should refer to the excessive accumulation of adipose tissue (triacylglycerols) [46].

Different methods are used to assess body mass, and BMI is a popular anthropometric indicator used for the overall assessment of body fat [44]. It is a simple and easy-to-use tool; however, it has some serious limitations. First, it cannot directly assess all aspects of body composition, such as visceral fat or fat breakdown. In addition, it does not differentiate between lean mass and body fat mass, which may result in a biased BMI score. Second, it does not consider such variables as gender, age, ethnicity, and leg length, and those parameters should also be considered when determining body fat mass [44,47]. It is worth stressing that women, in general, have a lower BMI than men, although their body fat mass relative to BMI is significantly greater (20.00% to 45.00%) [46]. Additionally, in the elderly, BMI is not an appropriate indicator of BFM due to the specific, age-related redistribution of abdominal fat [44].

Bioimpedance analysis constitutes a more reliable method of body fat assessment than BMI. Although the percent of body fat and body fat are predictable based on BMI score, the prediction accuracy is lower in the case of individuals with a BMI < 30.00. Frankenfield et al. reported that a significant number of people, despite having a BMI < 30.00, have obesity levels of body fat [48].

The above-mentioned facts strongly suggest not considering BMI solely in the analysis of factors affecting humoral response to the SARS-CoV-2 vaccine. Therefore, in our study, we decided to assess body fat using two methods independently: BMI and bioimpedance analysis.

Obesity is a recognized risk factor for severe COVID-19 outcomes [36,37]. Recent studies suggest that it is linked with approximately 5.00–10.00% higher risk for hospitalization per every kg/m^2^ higher BMI [49]. One possible explanation for these findings is that obesity causes low-grade chronic inflammation that may interfere with pathogens’ immune and thrombogenic responses. Moreover, excessive weight may disturb the proper functioning of the lungs, making obese people more susceptible to respiratory infections, whereby, what is worth stressing out, the most significant impact on lung functioning is the distribution of adipose tissue, and not BMI or weight in itself [50].

Numerous studies have confirmed that obesity is linked to greater susceptibility to infections [51]. Excessive body mass or fat gain may impair one’s immunity [51,52,53]. Additionally, the excess visceral adipose tissue may hinder and delay one’s immune response [54]. Adipose tissue expresses relatively high levels of the angiotensin-converting enzyme 2 (ACE2) receptor that SARS-CoV-2 uses to gain entry into cells [55]. However, how individuals with abdominal obesity respond to mRNA vaccines against SARS-CoV-2 has yet to be established. Inflammatory processes are involved in adipose tissue deposition and fasting. In humans and rodents, immune cells of the innate and adaptive immune systems have been detected in adipose tissue [56]. B cells play a versatile role in humoral immunity in both pro- and anti-inflammatory processes through antibody production, secretion of pro- and anti-inflammatory cytokines and chemokines, and antigen presentation [57].

Moreover, obesity is associated with other conditions that are independent risk factors and predictors of mortality from COVID-19, including diabetes, chronic cardiovascular, cerebrovascular, and pulmonary diseases [58]. As a result, obese people might have higher levels of a variety of immune-regulating proteins, including cytokines. In some COVID-19 patients, the immune response unleashed by cytokines can damage healthy tissue [59]. Earlier studies indicated the association between obesity and an impaired immune response to vaccines, which provoked concerns that SARS-CoV-2 vaccines might not be as effective in obese individuals [60,61,62,63,64,65]. However, based on the scientific evidence, Butsch et al. demonstrated that COVID-19 vaccines (BioNTech/Pfizer BNT162b2, Moderna mRNA-1273, Janssen/Johnson & Johnson Ad26.CoV2-S, AstraZeneca AZD-1222) are effective in obese individuals, and vaccine efficiency is not clinically significant in obese and non-obese subjects [66].

Furthermore, behavioral factors, such as smoking, can affect how individuals respond to vaccines. Toxic chemicals in cigarette smoke, such as carbon monoxide, nitrogen oxides, and cadmium, can influence the production of many immune mediators [67]. In addition, smoking impacts the immune system by affecting the immune B cells responsible for antibody production after vaccination. Despite various observations of the effects of tobacco smoke on B lymphocytes [68], smoking plays a harmful rather than beneficial role in most studies, as it attenuates the normal defensive function of the immune system. In a systematic review on COVID-19 risk factors, smoking was also likeliest correlated with a negative progression and adverse outcomes [69]. Smoking and smoking-related co-morbidities are likely associated with an impaired immune response to SARS-CoV-2.

In our study, smoking was one of the factors that negatively affected the production of IgG antibodies against SARS-CoV-2 in response to vaccination. Smokers, who accounted for 20% of the cohort, had a lower response in antibody titers at every measurement point than non-smokers. Similar observations were presented by Tsatsakis et al. The results of their research showed that non-smokers had higher titers than smokers [70]. Our findings also agree with a recent study by Parthymou et al., who, based on a 3-month observation after full vaccination against COVID-19, indicated that smoking is associated with a lower humoral response [71]. Likewise, those observations confirm Nomura et al.: in their study, three months after the second dose of the BNT162b2 vaccine, antibody titers were significantly lower in current smokers than in ex-smokers [72]. This phenomenon was also observed in reaction to other vaccines, i.a., against influenza. For example, MacKenzie et al., in their study, found that 50 weeks after immunization with a subunit vaccine, the longevity of the immune response in subjects who had no immunity before the vaccination was significantly lower in smokers in comparison to non-smokers [73].

The effect of cigarette smoking on immunity has been widely studied. It is usually associated with impaired function of immune cells, including B lymphocytes producing antibodies, often despite an increased number of B cells [68]. However, it must be stressed that when assessing the effect of smoking on the immune system response, other factors, such as smoking history or intensity, and genetic factors, must be taken under consideration. However, numerous findings demonstrate the detrimental effect of cigarette smoking on the maintenance of immunity after vaccination.

SARS-CoV-2, similar to other RNA viruses, shows a high capacity to mutate [31,74]. Newly-arising virus strains may differ regarding transmission capacity, virulence, symptoms triggered, disease course, and the immune response. Moreover, they may affect the efficacy and effectiveness of currently available vaccines against COVID-19 [75]. The emergence of novel variants, classified by the WHO as variants of concern: Delta (B.1.617.2) and Omicron (B. 1.1.529), confirms those observations [75,76]. In light of the facts mentioned above, a better understanding of factors affecting humoral response takes on new meaning. Studies show that the effectiveness of vaccination is slightly different and depends on virus variant. During our research, two variants of SARS-COV-2 were dominant in Poland: Alpha (B.1.1.7) and Delta (B.1.617.2). Studies have shown that the mRNA BNT162b2 vaccine is effective on Alpha and Delta variants, but there is a difference in the level of efficiency [77,78,79,80]. Considering the new variant SARS-COV-2 Omicron (B.1.1.529), most studies are still in progress. In small cohorts, the neutralization efficiency on the new variant shows lower neutralization against the Omicron variant than the Delta variant [81]. The impact of mutations emerging on new variants of SARS-COV-2 on vaccine efficacy is monitored. Most studies show that the immune response produced by vaccination is significantly effective against variants of the virus [77,78,79,80,81].

Our data provide important insights into the immune system’s dynamics of antibody production on the timeline in response to vaccination with the BNT162b2 vaccine. The SARS-CoV-2 virus-induced pandemic is still ongoing, and emerging subsequent virus variants pose a significant threat to public health. Therefore, elaboration of a strategy allowing the development and maintenance of high and long-lasting immunity after inoculation, especially in highly exposed groups, such as HCWs, should become an essential part of the COVID-19 management policy.

### Study Limitations

Attention should be paid to some limitations of this study. First, our study group was strongly feminized, which might have affected our analysis results. In addition, data regarding undergoing COVID-19 before the study started, i.e., its severity and course (asymptomatic/low-symptomatic/symptomatic)—factors that could impact the titer of SARS-CoV-2 IgG antibodies level and hence a longitudinal humoral response—was collected through self-reports, and thus could be the subject of bias.

## 5. Conclusions

Fully vaccinated convalescents are more likely to maintain an immune response than fully vaccinated volunteers without prior SARS-CoV-2 infection. Younger age and female gender are also associated with greater chances of developing long-lasting humoral responses after vaccination. Moreover, it is worth highlighting that increased muscle mass and decreased fat mass may positively affect long-term immunity following vaccination.

To the best of our knowledge, this is the first study assessing the impact of body composition (measured through electrical bioimpedance analysis), not BMI solely, on the immune response following vaccination against COVID-19. We presented the influence of adipose and muscle tissue levels on the immunization process after vaccination with the BNT162b2 vaccine. Our results highlight the importance of not considering body mass index exclusively when analyzing the variables affecting response to the SARS-CoV-2 vaccine.

A better understanding of factors affecting immune response after vaccination could result in introducing a more effective, tailored to particular groups’ conditions and immunological needs vaccination policy.

## Figures and Tables

**Table 1:** Demographic and clinical characteristics of the studied cohort of healthcare workers.

	*n*	%
Women	187	76.95
Men	56	23.05
Age over 60 years	33	13.58
Medical staff *	203	83.54
Administrative staff **	29	11.93
Diagnostic/sterilization staff ***	11	4.53
Chronic diseases	73	30.04
Smoking	45	18.52
Obesity (BMI ≥ 30.00)	44	18.11
Seropositivity before the first dose	62	25.51
Seropositivity eight months after the second dose	150	61.73
**Parameters**	**Mean**	**SD**
Age (years)	47.42	12.45
Metabolic age (years)	43.14	15.75
BFM (kg)	21.81	9.80
FFM (kg)	51.68	11.35
Body fat percentage (%)	28.92	8.03
Body fat range (level)	6.82	4.16
TBW (kg)	36.76	7.88
PMM (kg)	49.13	10.60
Bone tissue mass (kg)	2.61	0.53
Impedance (Ohm)	615.97	115.18
BMR (kJ)	6446.18	1371.61
IgG (pre-vaccination) (AU/mL)	14.16	40.14
IgG (peri-vaccination) (AU/mL)	147.08	200.96
IgG (post-vaccination—early FU) (AU/mL)	538.81	423.28
IgG (post-vaccination—late FU) (AU/mL)	26.01	40.57

Abbreviations: BMI—Body Mass Index, BFM—body fat mass, FFM—fat-free mass, TBW—total body water mass, PMM—predicted muscle mass, BMR—basal metabolic rate, IgG—immunoglobulin G, FU—follow-up, SD—standard deviation. * Medical staff: doctors, paramedics, nurses, medical guardian, physiotherapy technician/physiotherapist, electro radiology technician, pharmacist/pharmacy technician, psychologists, occupational therapists, dentist, students/interns. ** Administration staff: accountant, HR, clerk, inspector, health and safety specialist, public procurement specialist, secretary, medical registrar, hospital directors, medical statistician, cook, warehouse worker. *** Diagnostic/sterilization staff: diagnosticians, medical analytics technicians, medical sterilization technicians, cleaners.

**Table 2:** Change in the HCWs’ IgG titers before the vaccination with the BNT162b2 vaccine, during the immunization process and early and late follow-up.

IgG (AU/mL)	before the First Dose		before the Second Dose		Early FU		Late FU	
	*n*	%	*n*	%	*n*	%	*n*	%
<10.00 seronegative	181	74.49	22	9.05	2	0.82	93	38.27
≥10.00–50.00 (low response)	46	18.93	88	36.21	3	1.23	122	50.21
>50.00–100.00	7	2.88	30	12.35	3	1.23	19	7.82
>100.00–200.00	5	2.06	41	16.87	23	9.47	7	2.88
>200.00–350.00	4	1.65	36	14.81	64	26.34	1	0.41
>350.00 (high response)	0	0.00	26	10.70	148	60.91	1	0.41
≥10.00 seropositive (in all)	62	25.51	221	90.95	241	99.18	150	61.73

Abbreviations: IgG—immunoglobulin G; early FU—early follow-up (IgG titer 14 days after the second doses of the vaccine); late FU—late follow-up (IgG levels 8 months after the second dose of the vaccine).

**Table 3:** Influence of analyzed factors on the chances of a long-term immune response. One-way logistic regression analysis.

Variable	*n* (%)	*p* Value	OR	95% CI
Gender				
Male	56 (23.05)	0.45	1.28	0.68–2.38
Female	187 (76.95)		1.00	
Age (years)				
>60	33 (13.58)	0.00	0.30	0.14–0.64
<60	210 (86.42)		1.00	
Smoking				
Yes	45 (18.52)	0.11	0.58	0.30–1.12
No	198 (81.48)		1.00	
BMI (kg/m^2^)				
≥25.00	133 (54.73)	0.61	1.14	0.68–1.92
<25.00	110 (45.27)		1.00	
Obesity (BMI ≥ 30.00)				
Yes	44 (18.11)	0.77	1.10	0.56–2.17
No	199 (81.89)		1.00	
Seropositivity before the 1. dose of vaccine	62 (25.51)	0.00	8.64	3.54–21.06
BFM (kg)		0.96	1.00	0.97–1.03
FFM (kg)		0.08	1.02	1.00–1.05
TBW (kg)		0.06	1.03	1.00–1.07
PMM (kg)		0.06	1.03	1.00–1.05
Bone mass (kg)		0.09	1.56	0.93–2.64
Impedance (Ohm)		0.44	1.00	1.00–1.00
BMR (kJ)		0.06	1.00	1.00–1.00

Abbreviations: BMI—Body Mass Index, BFM—body fat mass, FFM—fat-free mass, TBW—total body water, PMM—predicted muscle mass, BMR—basal metabolic rate, OR—odds ratio, CI—confidence interval.

## Data Availability

The data used to support the findings of this research are available from the corresponding authors upon request.
